# The Influence of Health Literacy and Depression on Diabetes Self-Management: A Cross-Sectional Study

**DOI:** 10.1155/2016/3458969

**Published:** 2016-08-10

**Authors:** D. Maneze, B. Everett, C. Astorga, D. Yogendran, Y. Salamonson

**Affiliations:** ^1^South Western Sydney Local Health District, 59a Cumberland Road, Ingleburn, NSW 2565, Australia; ^2^School of Nursing and Midwifery, Western Sydney University, Locked Bag 1797, Penrith, NSW 2751, Australia; ^3^Centre for Applied Nursing Research, Locked Bag 7103, Liverpool, NSW 1871, Australia; ^4^Ingham Institute for Applied Medical Research, P.O. Box 3151, Liverpool, NSW 2170, Australia

## Abstract

Despite an increasing focus on health literacy in the clinical setting and in the literature, there is still ongoing debate about its influence on diabetes self-management. The aim of the study was to examine the relationships of sociodemographic, clinical, and psychological factors on health literacy and diabetes self-management. A cross-sectional survey was undertaken on 224 patients with type 2 diabetes at two diabetes centres in Sydney, Australia. Findings showed that people with low health literacy were more likely to (a) have lower educational attainment; (b) be migrants; and (c) have depressed mood. Unexpectedly, those who met HbA_1c_ threshold of good glucose control were more likely to have low health literacy. Predictors of low diabetes self-management included (a) younger age group (AOR: 2.58, 95% CI: 1.24–4.64); (b) having postsecondary education (AOR: 2.30, 95% CI: 1.05–5.01); (c) low knowledge of diabetes management (AOR: 2.29, 95% CI: 1.25–4.20); and (d) having depressed mood (AOR: 2.30, 95% CI: 1.30–4.06). The finding that depressed mood predicted both low health literacy and low diabetes self-management stresses the importance of screening for depression. Increasing people's understanding of diabetes self-management and supporting those with depression are crucial to enhance participation in diabetes self-management.

## 1. Introduction

The rapid rise in the global prevalence of diabetes lends urgency to the need for investigations beyond the walls of traditional factors such as poor nutrition, obesity, and sedentary behaviour. In 2013, diabetes was reported in 382 million people worldwide, a figure projected to increase by 55% to 592 million in 2035 [1]. Type 2 diabetes mellitus (T2DM) is the most prevalent form affecting 90% of adults with diabetes [2] and is increasingly being diagnosed in younger age groups [3]. While biochemical and clinical research is important, grassroots level sociocultural research is needed to understand the underlying sociodemographic and cultural environment which influences the self-efficacy of patients to perform the daily tasks of self-managing their chronic condition. For example, among migrants to many developed countries like Australia, acculturation to host culture, language and cultural barriers, and socioeconomic factors contribute to an increased incidence of lifestyle diseases, approximating that of the receiving country [4]. Migrants encounter many personal and systemic barriers in managing chronic conditions like diabetes [5, 6], which adds to the complexity of implementing self-management interventions in this population. Understanding how these factors interrelate and influence self-management is important to provide person-centred strategies to enhance the health of people living with diabetes.

Diabetes self-management (DSM) is considered an essential cornerstone of good diabetes control [7]. It is reported to reduce the level of glycated haemoglobin level (HbA_1c_), a clinical measure of adequate control, by as much as 37% [8]. Having a lower HbA_1c_ value (≤7% or ≤53 mmol/mol) reduces the likelihood of developing micro- and macrovascular complications over time [9]. Despite the increasing evidence that supports the benefits of DSM, uptake remains low, especially in culturally diverse populations [10, 11]. Among people with T2DM, knowledge deficit and understanding about diabetes and its complications have been found to be low in those with low health literacy [11], posing a barrier to DSM [12]. Given this association, improving health literacy, defined as* “the capacity to look for, process and understand health information to make informed decisions”* [13] seems an important priority to empower patients to self-manage their diabetes [14]. Paasche-Orlow and Wolf [15] postulated that the mechanisms contributing to poorer outcomes among those with low health literacy include low self-efficacy, lack of access to and utilisation of resources and services, and language and cultural issues in clinical encounters. It is important, however, to acknowledge that socioeconomic and demographic factors such as age, educational level, ethnocultural background, and having conditions that require complex care are underscoring limited health literacy [6, 10, 16]. Low levels of health literacy have been found to be common among patients who are from lower socioeconomic backgrounds and among migrants with limited English language proficiency, the elderly, and those with chronic diseases [17]. While some studies have found that low health literacy is associated with poor diabetes self-management, poor control, and more complications [12], the evidence regarding this association is inconsistent [18]. This could be due to other psychosocial and demographic factors that may affect health literacy and/or differences in measuring this construct [19].

Adding to the complications of suboptimal self-management is reduced psychological well-being [20]. This is a vicious cycle that may further disempower patients. For example, psychological comorbidity, like depression, contributes to lower self-care [21], which in turn leads to poorer health status leading to more depression and comorbidities which further reduce DSM [22].

The aim of the study was to examine the relationships between sociodemographic, clinical, and psychological factors and health literacy and its relationship with DSM within a culturally diverse urban population with T2DM. Specifically, we sought to investigate the relationship between health literacy and other factors influencing DSM. The hypotheses in this study were as follows:

(1) Self-management in patients with T2DM is associated with sociodemographic factors (age, gender, educational level, marital status, and country of birth), clinical factors (self-rated general health, HbA_1c_), and psychological factors (depression, confidence, knowledge, and health literacy).
(2) Health literacy in patients with T2DM is associated with sociodemographic factors (age, gender, educational level, marital status, and country of birth), clinical factors (self-rated general health, HbA_1c_), and psychological factors (depression, confidence, and knowledge).

## 2. Methods

### 2.1. Study Design and Setting

We used a cross-sectional design, patients with T2DM attending the diabetes outpatient clinics at two centres in South Western Sydney Australia. The study setting is a culturally diverse region with 52% of its population born overseas. Of these, 59% speak a language other than English at home and 13% are new arrivals, settling in Australia within the last five years [23]. Southwest Sydney is also one of the largest and most rapidly growing districts within the Sydney metropolitan area [24] with approximately 21% of the population in the low socioeconomic stratum [25] and only about 30% of its population completing secondary school. Unemployment rates are high, with a mean rate of 8% (range 5%–31%), ranking some of these suburbs (10 out of 38) among the most disadvantaged areas in Australia [25].

### 2.2. Participants and Recruitment

Using convenience sampling, participants were recruited between May and December 2015 from the outpatient diabetes clinics of two large centres in the South Western Sydney Local Health District (SWSLHD). Eligibility criteria included (1) age of 18 years and above; (2) being diagnosed with T2DM; (3) having HbA_1c_ test in the last two years recorded in their clinical file. Patients attending the outpatient clinics for their regular appointment with the diabetes educator, specialists, or dietician were identified and referred to the research team by the clinicians. One of the researchers then explained the purpose of study and sought consent from potential participants. Those who consented to participate in the study were asked to complete a questionnaire. Consent included access to participants' hospital records to retrieve their latest recorded clinical data including HbA_1c_, height, and weight. Researchers measured height and weight of participants to compute for their body mass index (BMI) after they have completed the questionnaire if this was not available in their clinical records.

### 2.3. Instruments

A pilot study was initially undertaken with 20 participants. The initial questionnaire consisted of 63 questions including items from five validated instruments: the English language acculturation scale [26], PHQ-2 depression scale [27], diabetes knowledge [28], diabetes self-efficacy [29], and diabetes self-management [30]. Results of the pilot testing indicated that participants found the questionnaire to be too complex and lengthy, including those whose first language was English. Following discussion with the research team, a consensus was reached to simplify the questionnaire and reduce the survey to only include 34 items. These were items related to demographic and clinical characteristics, three brief validated measures, namely, the (a) 3-item Health Literacy Scale [31]; (b) 2-item PHQ-2 to assess depressed mood and anhedonia [27]; and (c) 16-item Diabetes Self-Management Scale [30]. As single item questions have been found to be as valid and reliable as multiple-item scales, particularly when constructs that are being measured are fairly homogenous [32, 33] the two standardised scales that measured diabetes self-efficacy and diabetes knowledge were replaced with two single items; namely,* “In a scale of 1 to 10 (1 being not confident to 10 being very confident), how confident are you that you will be able to manage your diabetes?”* and* “In a scale of 1 to 10 (1 being very poor to 10 being excellent), how do you rate your knowledge about diabetes?”* Subjective assessment of perceived overall health was likewise assessed with a single question:* “In general, how would you describe your general health?”* with a five-point Likert scale response,* excellent, very good, good, fair, and poor*.

Cronbach's alpha was calculated for each validated instrument used. This is a measure of the extent to which the items in the questionnaire consistently assess the same idea or concept [34]. The internal consistency is expressed as a numerical value between 0 and 1 with scores between 0.70 and 0.90 indicating good correlation among items in the questionnaire [35].

#### 2.3.1. Brief Health Literacy Scale

Health literacy was evaluated using the 3-item Health Literacy Scale [31] and included the following: (1)* How often do you have problems learning about your medical condition because of difficulty understanding written information?* and (2)* How confident are you filling out forms by yourself?* and (3)* How often do you have someone help you read hospital materials*? Each item was rated with a 5-point Likert scale with lower scores indicating lower health literacy.

#### 2.3.2. Depression Scale

The 2-item Patient Health Questionnaire (PHQ-2) [27] was used to assess anhedonia and depressed mood over a 2-week period. This 4-point Likert scale has been used extensively to determine the presence of depression, with higher scores indicating the presence of depression [36]. A cut-off aggregate score of 2 has been found to have high sensitivity in detecting major depressive disorder (92.7%) and any depressive disorder (80.4%), with specificity of 73.7% and 80.4%, respectively [27].

#### 2.3.3. Diabetes Self-Management Scale

The 16-item Diabetes Self-Management Questionnaire (DSMQ-16) [30] was used in this study because of its brevity relative to other related scales. More importantly it had significantly stronger correlation with HbA_1c_ which is an important measure of diabetes control.

#### 2.3.4. Glycated Haemoglobin (HbA_1c_)

The glycated haemoglobin level or HbA_1c_ is recommended in the monitoring of glucose control as it reflects the average blood glucose level over three months and has a good correlation with diabetes complications [37]. A cut-off value of 7% has been recommended to indicate good control [37, 38].

### 2.4. Analysis

Sample size calculation for the outcome variable was based on low DSM rate of 50%. Taking into account the 11 sociodemographic, clinical, knowledge, and psychological predictor variables as listed in the hypothesis and using the sample size calculation based on Peduzzi et al. [39] of *N* = 10*k*/*p* (where *N* is the minimum number of cases needed, *k* is the number of predictor variables, and *p* is the proportion of low DSM rate), the minimum sample size required was 220.

We used SPSS version 23 software [40] for all data analysis. Frequencies and percentages were computed for categorical variables, and mean, median, and standard deviation and interquartile range were computed for continuous variables. As none of the continuous variables were normally distributed, age, duration of diabetes diagnosis, BMI, medical comorbidities, confidence, knowledge, health literacy, and DSMQ-16 scores were dichotomised at the median. However, the PHQ-2 score was dichotomised at 2 to represent “not depressed” (0-1) and “depressed” (2–6) to be consistent with the high sensitivity of this cut-off shown in previous studies [36]. While dichotomisation of variables may have the disadvantage of loss of analytical power and some important information, it has the benefits of reducing the variability in a skewed data and consequently the random error, making the results more accurate [41]. In addition, it simplifies the results and thus presents findings that are easily understandable to a wide range of audience [42]. Data in this study was skewed with a high variability in the responses. Furthermore, corrective logarithmic transformation calculations performed did not produce findings dissimilar to the dichotomised results obtained.

The Chi-square test was used to assess relationships between two categorical variables, and logistic regression analysis (forward conditional method, with listwise deletion of cases with missing data [43]) was used to identify predictors of depression and predictors of DSM. The variables included in these regression analyses were demographic, clinical, and psychological characteristics of participants as previously described in the hypotheses.

## 3. Results

In total, 275 patients who met the inclusion criteria were approached. Of these 11 refused to participate and 40 were excluded from the final analysis as they were not able to complete the questionnaire and/or they did not have a recorded HbA_1c_ in the last two years. Cronbach's alpha for the following instruments used in the study showed good item correlation and internal consistency: Brief Health Literacy Scale (*α* = 0.83); Depression Scale (PHQ-2) (*α* = 0.88); and the Diabetes Self-Management Scale (DSM-16) (*α* = 0.79).

The demographic profile of our sample approximated the statistical profile of the study setting. Of the 224 participants included in the final analysis, 56% were born overseas and 7% were newly arrived migrants (less than 5-year duration of stay in Australia) with 40% speaking a language other than English at home. Nineteen percent (19%) of the sample had postsecondary schooling.  Table 1 shows the clinical, knowledge, and psychological characteristics of participants. Although the overall health literacy score was high (median: 10; range: 0–12), the overall diabetes knowledge score was lower (median: 7, range: 0 to 10). While the overall DSM-16 score was high (median: 35, range: 7 to 47), 61% of the sample were obese (BMI: ≥30 kgm^2^), and 81% had an HbA_1c_ over 7% with 30% having more than two comorbidities. Forty-seven percent (47%) of the participants rated their general health as fair to poor. Fifty percent of the participants had a score of 2 or more in the PHQ-2 suggesting the presence of depressed mood or anhedonia [27, 36]. Those who had PHQ-2 score more than 2 were also found to have longer duration of diabetes diagnosis (more than 10 years), more comorbidities (more than 2), lower confidence, and less DSM behaviours.

### 3.1. Group Comparisons of Low and High Health Literacy

Using the median score of 10 as the cut-off for the Brief Health Literacy scale, group comparisons of sociodemographic, clinical, and knowledge and psychological factors were computed using the Chi-square test. As shown in Table 2, those who were older, had up to primary schooling, were overseas-born, were less confident about diabetes management, and had PHQ-2 score ≥ 2, had low health literacy. Surprisingly, those with HbA_1c_ > 7%, indicating poor control, were more likely to have high health literacy (*p* = 0.047).

### 3.2. Predictors of Low Health Literacy

Using forward stepwise logistic regression analysis, four variables emerged as independent and significant predictors of low health literacy: (i) education; (ii) country of birth; (iii) glucose control as measured by HbA_1c_; and (iv) depression. In relation to educational attainment, those with up to primary schooling were more likely to have low health literacy (AOR: 3.12, 95% CI: 1.17 to 8.30); conversely, those with postsecondary school were less likely to have low health literacy (AOR: 0.35, 95% CI: 0.16 to 0.74).  Table 3 also shows that those born overseas were over two times (AOR: 2.17, 95% CI: 1.21 to 3.91) more likely to have low health literacy; similarly, those who were depressed were also over two times (AOR: 2.01, 95% CI: 1.12 to 3.59) more likely to have low health literacy. Unexpectedly, those with good glucose control, as indicated by HbA_1c_ of up to 7%, had low health literacy (AOR: 0.41, 95% CI: 0.29 to 0.90). These four variables explained approximately 19% of the variance (Nagelkerke *R* ^2^ = 0.191), and Hosmer-Lemeshow goodness-of-fit statistics was not significant (Chi-square = 2.937, df = 7, and *p* = 0.891), indicating good model fit.

### 3.3. Predictors of Low Diabetes Self-Management

Forward stepwise logistic regression analysis was likewise used to determine predictors of DSM, using the median of up to 35 as the cut-off score. Four variables emerged as independent and significant predictors of low DSM: (i) younger age group (≤60 years); (ii) having postsecondary schooling; (iii) low diabetes management knowledge score (≤7), and (iv) being depressed (PHQ-2: ≥2). The magnitude of the adjusted odds ratios was similar for all four predictor variables, ranging from 2.30 to 2.58, explaining approximately 17% of the variance (Nagelkerke *R* ^2^ = 0.166). The Hosmer-Lemeshow goodness-of-fit statistics was not significant (11.635, df = 7, *p* = 0.113), indicating good model fit (Table 4).

## 4. Discussion

In the current study, those with only primary school education, migrants, and those who reported depressed mood were more likely to have low health literacy. The relationship between education and health literacy has previously been reported [44]; while this was not an unexpected finding it was encouraging to find that participants with secondary schooling and above reported adequate health literacy. Further analysis of those with primary school education revealed that they were also more likely to be older (79%) and overseas-born (70%), which has important implications for targeting this group considering the demographic profile of the current study setting and its being a major area for immigrant settlement in Australia [23]. This is particularly important given that migrants have a disproportionately high prevalence of diabetes [45] and face a number of barriers such as limited English language proficiency, access issues, cultural beliefs, and socioeconomic factors that could have direct and indirect effects on health literacy and DSM [5, 45–47]. Compared with Australian-born participants, migrants in this study had significantly lower confidence in their ability to manage their diabetes (*p* = 0.019). Culturally tailored resources and lifestyle interventions addressing these barriers including fostering problem-solving skills, cultivating motivation by setting appropriate goals, and consistent follow-up could be important tools to build confidence for self-managing diabetes in this population.

Depression has been found to affect diabetes control through both physiological pathways [48], effects of treatment [49], and/or increasing demand for psychological and behavioural tasks involved in DSM [50]. Our study confirmed the finding that depressed mood and anhedonia are associated with low self-efficacy in carrying out DSM. The PHQ-2 is sensitive, quick, and easily administered in a busy clinic setting which could allow for referral for psychological support. Given the negative influence of depression on diabetes control through several mechanisms, an important recommendation from this study would be that clinicians consider screening all patients who attend diabetes clinics for depression using the PHQ-2.

An unexpected outcome of our study was that poorer glucose control, as demonstrated by high HbA_1c_, was correlated with higher health literacy. This may be explained by two factors: the Health Literacy Scale used in this study measured general health literacy rather than health literacy specific to diabetes and therefore may not be suitable for the sample in this study. For example, one of the questions in this tool* “How often do you have someone help you read hospital materials*?*”* was answered by a number of participants with* “never, because there was never anybody there to help me, I had to read them by myself,”* which reflected lack of support rather than a high level of health literacy. Secondly, having high health literacy may not necessarily translate into self-management actions that could result in better biochemical diabetes control. This contention is supported by the findings in this study that those who were highly educated had high health literacy but reported low DSM however; those who had higher diabetes knowledge score had higher DSM. A study on English-speaking adults with type 2 diabetes likewise found that health literacy (measured using S-TOFHLA) was not associated with HbA_1c_ or with the presence of diabetes complications [18]. In contrast, Schillinger et al. [12] found an association between low health literacy, poor diabetes control, and retinopathy in an ethnically diverse population.

Older participants in this study practiced more DSM although they had lower health literacy, perhaps because of heightened awareness of mortality whereby health becomes a main concern. It could also be that older participants spent more time engaging in DSM tasks as they had less external competing priorities compared with younger and more educated participants who, presumably, had job demands and family concerns which took priority over DSM. This study found no significant correlation between health literacy and DSM; however, it was lack of knowledge about diabetes specifically that predicted lower DSM.

A number of variables measured in this study were self-assessed constructs that were useful in illuminating the perceptions of participants regarding their resources in effecting DSM. For example, despite more than half of the participants' rating their health as good (53%) and reporting adequate self-management (54%), objective measurements of BMI and HbA_1c_ showed that a high number (61% and 81%, resp.) of participants were obese and/or had poorly controlled diabetes. This discordance between what participants perceived as “good” DSM and clinical parameters of good control is also consistent with previous studies of people with diabetes and other chronic conditions. For example, in a sample of rural Taiwanese residents Huang et al. [51] found that those who had HbA_1c_ ≥ 7%, indicating a poor level of control, assessed their health as good [51]. Large population-based studies have also demonstrated a “disconnect” between perceived and actual health in approximately one out of five individuals, with younger age, ethnicity (non-Hispanic Blacks), and higher socioeconomic status predicting this disconnect [52]. This discrepancy between self-perceived health status and objective measures of diabetes control is likely to have clinical implications for DSM education as improving the convergence between perceived and actual health may help promote self-management and, ultimately, improve health outcomes. It is therefore important for health professionals to stress the importance of maintaining a healthy weight and achieving optimal HbA_1c_ in patient diabetes education programs.

This study has several limitations. The participants were sampled from a cohort that is already accessing the diabetes clinics of two major centres in the region. This may not be representative of the general population with type 2 diabetes. Secondly, the study was cross-sectional and, given the chronicity of diabetes, a longitudinal study may have been more useful in assessing the effect of the variables under examination in relation to self-management over a period of time. Thirdly, the use of Chew's brief measure of health literacy may not accurately have reflected the level of health literacy in our participants. The tool had ceiling effects which may not be due to high health literacy. Another limitation of the study was the lack of recent (within the last three months) HbA_1c_ level, as some of the HbA_1c_ results used in this study were taken within the last two years (2014–2016) and, therefore, may not have been contemporaneous with data collection. Finally, as with all studies that collect data using self-report measures, social desirability bias may have impacted on these findings and, given the discrepancy between self-reported health and HbA_1c_, this seems possible. Notwithstanding these limitations, this study presented findings that refute the relationship between health literacy and DSM in a culturally diverse urban population.

## 5. Conclusion

Sociocultural research exploring the factors affecting DSM is important to determine areas that may be amenable to implementing cost-effective interventions. In culturally diverse populations with T2DM, while sociocultural factors are determinants of health literacy, this study has demonstrated that it was not health literacy per se but having knowledge specific to diabetes that was more important in predicting the practice of DSM behaviours. Addressing the discordance in perception of health and objective measures of diabetes control in DSM education may improve patient compliance and monitoring. Importantly, the finding that depression was a significant predictor of both low health literacy and low DSM underscores the need for clinicians to screen for depression to ensure that people with T2DM are provided with appropriate support which in turn may enable them to engage in self-managing their condition.

## Figures and Tables

**Table 1:** Characteristics of T2DM participants (*n* = 224).

Characteristics	
*Sociodemographic*	
Age, median (*IQR*) years (range: 22–90)	60 (17)
Sex: male, *n* (%)	119 (53)
Marital status: with partner, *n* (%)	147 (66)
Highest educational attainment^*∗*^	
(i) Up to primary schooling, *n* (%)	33 (15)
(ii) Secondary schooling (years 7 to 12, TAFE, trade), *n* (%)	146 (65)
(iii) More than secondary schooling (postgraduate), *n* (%)	42 (19)
Country of birth: overseas-born	125 (56)

*Clinical characteristics*	
Self-rated health: fair or poor, *n* (%)	98 (47)
Duration of diabetes diagnosis: median (*IQR*) years (range: 0–46)	10.0 (11)
Body mass index (BMI): median (*IQR*) (range: 19.0–64.5)	32.4 (11)
HbA_1c_: median (*IQR*) (range: 4.8–14.0)	8.4 (3)
Number of medical comorbidities: median (*IQR*) (range: 0–7)	2.0 (2)

*Knowledge and psychological factors*	
Depression: PHQ-2 score: median (*IQR*) (range: 0–6)	1.5 (3.0)
Confidence in managing their diabetes: median (*IQR*) (range: 1–10)	8.0 (3.0)
Knowledge about diabetes: median (*IQR*) (range: 0–10)	7.0 (3.0)
Health literacy score: median (*IQR*) (range: 0–12)	10.0 (−7.0)
DSMQ-16 score: median (*IQR*) (range: 7–47)	35.0 (12)

^*∗*^Missing data.

**Table 2:** Group comparison of high and low health literacy levels by participant characteristics.

Characteristics	Low health literacy (≤10)	High health literacy (>10)	Unadjusted odds ratio	*p*
*Sociodemographic*				
Age: <60 years, *n* (%)	56 (44)	57 (58)	0.58 (0.34–0.98)	0.042
Sex: male, *n* (%)	65 (52)	54 (55)	1.15 (0.68–1.96)	0.601
Marital status: with partner, *n* (%)	86 (68)	61 (62)	0.77 (0.44–1.34)	0.348
Highest educational attainment				
(i) Up to primary schooling	27 (22)	6 (6)	0.24 (0.09–0.61)	<0.001
(ii) Secondary schooling	81 (66)	65 (66)	1.09 (0.63–1.91)	
(iii) More than secondary schooling	15 (12)	27 (28)	2.81 (1.40–5.66)	
Country of birth: overseas-born, *n* (%)	80 (64)	45 (46)	0.49 (0.29–0.84)	0.009

*Clinical characteristics*				
Self-rated health: fair or poor, *n* (%)	111 (90)	88 (90)	0.97 (0.41–2.32)	0.946
HbA_1c_: high (>7%), *n* (%)	96 (76)	85 (87)	2.04 (1.00–4.17)	0.047

*Knowledge and psychological factors*				
Confidence diabetes management: low (up to 8), *n* (%)	94 (75)	60 (61)	1.86 (1.05–3.29)	0.032
Knowledge about diabetes: low (up to 7), *n* (%)	83 (66)	53 (54)	1.64 (0.95–2.82)	0.073
Psychological status, depressed (PHQ-2: ≥2)	71 (56)	41 (42)	0.56 (0.33–0.95)	0.031
DSMQ-16 score: low (up to 35), *n* (%)	64 (51)	57 (58)	0.74 (0.44–1.26)	0.283

**Table 3:** Predictors of low health literacy in T2DM patients (*n* = 224).

Variables	Adjusted odds ratio (95% CI)	Std error (SE)	*p*
(i) Highest educational attainment (reference: secondary schooling)			
(a) Up to primary schooling	3.12 (1.17 to 8.30)	0.50	0.023
(b) More than secondary schooling	0.35 (0.16 to 0.74)	0.39	0.006
(ii) Country of birth: overseas-born	2.17 (1.21 to 3.91)	0.30	0.010
(iii) Poor glucose control (HbA_1c_: >7%)	0.41 (0.19 to 0.90)	0.40	0.026
(iv) Psychological status, depressed (PHQ-2: ≥2)	2.01 (1.12 to 3.59)	0.30	0.019

CI denotes confidence interval.

Nagelkerke *R* ^2^ = 0.191.

Hosmer-Lemeshow goodness-of-fit for the model: Chi-square = 2.937, df = 7, and *p* = 0.891.

**Table 4:** Predictors of low diabetes self-management in T2DM patients (*n* = 224).

Variables	Adjusted odds ratio (95% CI)	Std error (SE)	*p*
(i) Age: ≤60 years	2.58 (1.25 to 4.64)	0.30	0.001
(ii) Highest educational attainment: more than secondary schooling	2.30 (1.05 to 5.01)	0.40	0.037
(iii) Diabetes knowledge: less than ≤7	2.29 (1.25 to 4.20)	0.31	0.007
(iv) Psychological status, depressed (PHQ-2: ≥2)	2.30 (1.30 to 4.06)	0.29	0.004

CI denotes confidence interval.

Nagelkerke *R* ^2^ = 0.166.

Hosmer-Lemeshow goodness-of-fit for the model: Chi-square = 11.635, df = 7, and *p* = 0.113.
